# The Story of the Insane from Year to Year

**Published:** 1898-05-21

**Authors:** 


### THE STORY OF THE INSANE FROM YEAR TO YEAR.

### Tenth Series.

### SOMERSET COUNTY ASYLUM AT WELLS.

During the year 1896 the numbers increased from 885 to 900. There were 201 first admissions, 37 readmissions, 87 recoveries, 14 discharged relieved, and 109 deaths. The recoveries stand at 38·1 per cent. on the admissions, and the deaths at 11·1 per cent. on the average number resident. Of the deaths 31 were caused by cerebral or spinal diseases, 18 by consumption, 4 by pneumonia, 1 by dysentery, and 1 by enteritis. Of the admissions 24 owed their insanity to various moral causes, 10 to drink, and 21 to hereditary influence; but as 77 are stated to be of unknown cause, the table is not of much use. Seven of the year's admissions figure as being due to religion. Although the number of patients in the asylum has increased in ten years by 279, Dr. Wade shows that this is not owing to any alarming increase of insanity in the county, but that the factors of this increase have been the removal of lunatics from workhouses, improved care of patients causing longer lives, and a greater willingness to send patients to asylums now than was the case some years ago. Dr. Wade is to be congratulated as regards his staff. He has no charge attendant with less than fourteen years' service, and of his charge nurses only one has less than five years' service. The committee say that "Dr. Wade has actively devoted himself to the discharge of his onerous duties." The weekly charge to the Unions is 9s. 4d. per head.

The following extract from the New York *Medical Record* is a neat sarcasm upon hospital and dispensary abuse as it appears at the other side of the Atlantic: "Dispensary practice has been affected by the war. In a down-town district a falling-off in attendance at one of the dispensaries was noted for a few days, which could not be accounted for until it was found there was a 'run' on a neighboring bank, and the patients were busy drawing out their deposits."

